# Impact of Serum GDF-15 and IL-6 on Immunotherapy Response in Cancer: A Prospective Study

**DOI:** 10.3390/cancers16244146

**Published:** 2024-12-12

**Authors:** Orhun Akdogan, Sena Turkmen, Galip Can Uyar, Kadriye Bir Yucel, Busra Tufekci, Fatih Gurler, Ozan Yazici, Nuriye Ozdemir, Ahmet Ozet, Cengiz Karakaya, Osman Sutcuoglu

**Affiliations:** 1 Department of Medical Oncology, Gazi University, 06560 Ankara, Türkiye; fatihgurler@gazi.edu.tr (F.G.); ozanyazici@gazi.edu.tr (O.Y.); nuriyeozdemir@gazi.edu.tr (N.O.); ahmetozet@gazi.edu.tr (A.O.); 2 Department of Medical Biochemistry, Gazi University, 06560 Ankara, Türkiye; senatrkmen@gmail.com (S.T.); karakayac@gazi.edu.tr (C.K.); 3 Department of Medical Oncology, Ankara Etlik City Hospital, 06170 Ankara, Türkiye; galipcan.uyar@saglik.gov.tr (G.C.U.); kadriyebiryucel@gazi.edu.tr (K.B.Y.); sutcuogluo@gmail.com (O.S.); 4 Department of Internal Medicine, Gazi University, 06560 Ankara, Türkiye; busra.tufekci@gazi.edu.tr

**Keywords:** Growth Differentiation Factor 15 (GDF-15), Interleukin-6 (IL-6), immunotherapy response

## Abstract

Background: Immunotherapy has transformed cancer treatment; however, predicting treatment response remains challenging. Serum biomarkers can help identify patients who are most likely to benefit from immunotherapy. Objective: This study evaluates the relationship between serum growth differentiation factor 15 (GDF-15) and interleukin-6 (IL-6) levels and treatment outcomes in cancer patients undergoing second-line immunotherapy. Methods: We conducted a prospective observational study involving 85 patients with non-small-cell lung cancer (NSCLC), renal cell carcinoma (RCC), or malignant melanoma treated with nivolumab. The baseline serum levels of GDF-15 and IL-6 were measured by using ELISA kits. The primary endpoints were progression-free survival (PFS) and overall survival (OS), with cachexia as a secondary outcome. Results: Elevated GDF-15 levels were significantly associated with shorter PFS (HR: 0.55, 95% CI: 0.32–0.96, *p* = 0.032) and OS (HR: 0.47, 95% CI: 0.25–0.90, *p* = 0.020). Higher IL-6 levels correlated with shorter PFS, though statistical significance was not achieved. Additionally, high GDF-15 levels were linked to increased cachexia incidence (*p* = 0.037). Conclusion: Our findings indicate that GDF-15 could serve as a prognostic biomarker for immunotherapy response and may also be a target for cachexia management. Further studies should explore its potential to guide clinical decision making in oncology.

## Simple Summary

Immunotherapy has significantly changed cancer treatment, yet predicting which patients it will benefit remains challenging. This study aimed to explore the role of two serum biomarkers, growth differentiation factor 15 (GDF-15) and interleukin-6 (IL-6), in determining the outcomes of patients receiving immunotherapy. By analyzing 85 patients with advanced cancers such as lung, kidney, and melanoma, we discovered that high levels of GDF-15 were associated with worse survival outcomes and a higher likelihood of cancer-related cachexia, a condition causing severe weight and muscle loss. Although IL-6 also showed potential as a biomarker, its impact was less pronounced. These findings suggest that GDF-15 could serve as a useful biomarker to guide treatment decisions and help identify patients who might benefit the most from immunotherapy. This research study highlights the importance of using biomarkers to optimize cancer care and improve patient outcomes.

## 1. Introduction

Immunotherapy has demonstrated effectiveness across several cancer types; however, it remains costly and is not universally effective for all patients [1]. Current clinical practice aims to identify patients likely to benefit from treatment through biomarkers such as PD-L1 expression, nutritional status, and Eastern Cooperative Oncology Group (ECOG) performance scores [2,3,4,5,6]. Despite these efforts, there is still a need for a dynamic serum biomarker that can be easily measured and used to predict treatment response.

Growth differentiation factor 15 (GDF-15), a member of the transforming growth factor-beta (TGF-beta) superfamily, is increasingly recognized for its role in immune modulation and as a marker of cancer-related cachexia [7]. GDF-15 contributes to immune evasion by inhibiting T-cell activity within the tumor microenvironment, potentially diminishing the efficacy of immune checkpoint inhibitors (ICIs) [7,8]. Additionally, GDF-15 plays a critical role in the development of cancer cachexia, a multifactorial syndrome associated with significant morbidity and mortality in cancer patients [9].

Similarly, interleukin-6 (IL-6), a pro-inflammatory cytokine, has been studied as an immune biomarker, but its role in modulating responses to ICIs remains to be fully elucidated [9,10]. Elevated IL-6 levels have been associated with poorer outcomes in cancer patients, but its specific role in modulating responses to ICIs remains unclear. Several studies have examined the prognostic value of IL-6 in cancer therapy, yet its relationship with biomarkers such as GDF-15 has not been fully explored [11].

Although both biomarkers have been studied independently, the relationship between serum GDF-15 and IL-6 levels in predicting immunotherapy response has not been comprehensively evaluated. Furthermore, considering the global disparities in access to immunotherapy, particularly in low- and middle-income countries, identifying biomarkers like GDF-15 to prioritize patients most likely to benefit from these therapies is of both clinical and economic significance [12].

This study aims to evaluate the relationship between serum GDF-15 and IL-6 levels and their impact on immunotherapy response in cancer patients. Specifically, it assesses the association of GDF-15 with cancer-related cachexia and its potential role as a prognostic marker. By exploring the correlation between these biomarkers and treatment outcomes, this study seeks to provide new insights into their prognostic value and to contribute to the growing body of literature on biomarkers in cancer immunotherapy.

## 2. Methods

### 2.1. Study Design

This prospective observational cohort study was conducted between May 2023 and February 2024 at two tertiary care centers in Ankara, Turkey: Gazi University Medical Oncology Clinic and Etlik City Hospital Oncology Clinic. The objective was to assess the association of serum growth differentiation factor 15 (GDF-15) and interleukin-6 (IL-6) levels with immunotherapy response and cancer-related cachexia in patients diagnosed with non-small-cell lung cancer (NSCLC), renal cell carcinoma (RCC), and melanoma.

### 2.2. Study Population

The study population consisted of patients who were initiated on second-line immunotherapy with nivolumab between May 2023 and February 2024 and who had been diagnosed with NSCLC, RCC, or malignant melanoma. Due to national reimbursement policies in Turkey, patients receiving nivolumab were required to have undergone at least one prior line of systemic therapy, as first-line immunotherapy is not covered by insurance. Therefore, all patients included in the study had previously received chemotherapy or tyrosine kinase inhibitors (TKIs), and nivolumab was administered as a second-line treatment.

Eligible patients were aged 18 years or older, with a histologically confirmed diagnosis of NSCLC, RCC (clear cell subtype only), or cutaneous malignant melanoma. All patients were immunotherapy-naive and were not receiving concurrent chemotherapy or tyrosine kinase inhibitors. Patients with small-cell lung cancer, non-clear cell RCC subtypes, or non-cutaneous melanoma were excluded. Additionally, patients with acute infections, acute coronary syndrome, or comorbid conditions that could potentially affect serum GDF-15 or IL-6 levels—such as chronic inflammatory diseases, severe liver or kidney dysfunction, or autoimmune disorders—were also excluded, as these conditions are known to influence biomarker levels [13,14,15].

In total, 97 patients met the initial eligibility criteria and were enrolled in the study. However, 12 patients were excluded due to irregular follow-up visits, resulting in a final cohort of 85 patients included in the analysis. Excluded patients were primarily those unable to comply with regular follow-up visits or those who withdrew consent during the study period. Regular follow-up was mandatory to evaluate treatment-related toxicities and cancer-associated cachexia. Cachexia was defined according to the international consensus guidelines as a multifactorial syndrome characterized by an ongoing loss of skeletal muscle mass (with or without fat loss) that cannot be fully reversed by conventional nutritional support, leading to progressive functional impairment. In this study, cancer-associated cachexia was specifically identified by an unintentional weight loss of ≥5% over a six-month period or a body mass index (BMI) of less than 20 kg/m^2^ in combination with ≥2% weight loss, as per the diagnostic criteria established by Fearon et al. [16]. Other adverse events related to nivolumab therapy were also documented.

### 2.3. Outcomes

The primary outcomes of the study were progression-free survival (PFS)—defined as the time from immunotherapy initiation to either disease progression or death from any cause—and overall survival (OS), defined as the time from treatment initiation to death from any cause. Secondary outcomes included the occurrence of cancer-associated cachexia, defined by international consensus criteria as unintentional weight loss of ≥5% over six months or a body mass index (BMI) below 20 kg/m^2^ combined with ≥2% weight loss.

### 2.4. Sample Collection and Biomarker Analysis

Blood samples were collected from all participants before the initiation of nivolumab therapy. To minimize the potential effects of diurnal variations in GDF-15 levels, samples were standardized by collecting them in the early morning (08:00–09:00) while patients were in a fasting state. Serum was separated by centrifugation at 3000 rpm for 10 min and subsequently stored at −80 °C until further analysis. Serum levels of GDF-15 and IL-6 were measured by using enzyme-linked immunosorbent assay (ELISA) kits (R&D Systems, Minneapolis, MN, USA) at the Biochemistry Laboratory of Gazi University. All assays were performed in duplicate, and the average values were used for analysis. The intra-assay coefficient of variation (CV) for both GDF-15 and IL-6 was less than 10%, ensuring assay reliability.

### 2.5. Ethical Considerations

The study was initiated following approval from the Ethics Committee of Ankara Etlik City Hospital (approval number AESH-BADEK-630). Written informed consent was obtained from all participants prior to inclusion in the study. The study was conducted in accordance with the ethical standards of the Declaration of Helsinki, as well as local regulations on clinical research. The confidentiality of patient data was strictly maintained, and participants were assured that their involvement in the study would not affect their treatment.

### 2.6. Statistical Analysis

All statistical analyses were conducted by using SPSS software, version 25.0 (IBM Corp., Armonk, NY, USA). Descriptive statistics were used to summarize baseline patient characteristics, serum biomarker levels, and clinical outcomes. Spearman rank correlation analysis was performed to assess the relationship between serum biomarker levels and progression-free survival. Kaplan–Meier survival curves were constructed to estimate PFS and OS, with comparisons between survival curves performed by using the log-rank test. Cox proportional hazards regression models were utilized to evaluate the association between serum GDF-15 and IL-6 levels and clinical outcomes, including PFS, OS, and the development of cachexia, while adjusting for potential confounders such as age, sex, cancer type, and ECOG performance status. Hazard ratios (HRs) and 95% confidence intervals (CIs) were reported. A *p*-value of <0.05 was considered statistically significant, and variables with *p*-values < 0.10 in univariate analyses were included in the multivariate model.

Although the study investigated the potential predictive value of serum GDF-15 and IL-6 levels, no standardized cutoff values exist in the literature for predicting progression. Therefore, patients were stratified into two groups based on the median values of these biomarkers. ROC analysis was not conducted to determine cutoffs, as both PFS and OS were assessed as continuous variables, making median-based grouping appropriate for this exploratory analysis.

## 3. Results

### 3.1. Patient Characteristics

Initially, 97 patients were enrolled, but 12 were excluded, resulting in a final cohort of 85 patients, including 58 males (68%) and 27 females (32%). The median age of the patients was 64.5 years (range: 34–80 years). The majority of patients had non-small-cell lung cancer (NSCLC) (*n* = 50, 59%), followed by renal cell carcinoma (RCC) (*n* = 23, 27%) and malignant melanoma (*n* = 12, 14%). The median serum GDF-15 level was 26.4 ng/mL (range: 14.7–798.9 ng/mL), and the median IL-6 level was 20.0 pg/mL (range: 1.0–183.3 pg/mL) (Table 1).

### 3.2. Spearman Correlation Analysis

Correlation analysis was conducted to examine the relationships between serum GDF-15 and IL-6 levels and median progression-free survival (mPFS). The results demonstrate a significant negative correlation between GDF-15 levels and mPFS (ρ = −0.416, *p* < 0.001), indicating that higher GDF-15 levels were associated with shorter mPFS. Similarly, IL-6 levels were also negatively correlated with mPFS (ρ = −0.257, *p* = 0.018), suggesting that higher IL-6 levels were associated with shorter mPFS, although the strength of this correlation was weaker compared with GDF-15 (Table 2).

No significant correlation was found between GDF-15 and IL-6 levels (ρ = −0.063, *p*: 0.569), indicating that the levels of these two biomarkers were independent of each other in this cohort.

### 3.3. Grouping Based on Median GDF-15 and IL-6 Levels

To further explore the prognostic value of serum biomarker levels, we stratified patients into groups based on the median values of GDF-15 and IL-6. Patients with GDF-15 levels below the median (26.4 ng/mL) were assigned to the low-GDF-15 group (*n* = 43), while those with levels above the median were placed in the high-GDF-15 group (*n* = 42). Similarly, patients were divided into low- and high-IL-6 groups by using the median IL-6 level (20.0 pg/mL). The baseline characteristics, including cancer type, gender, ECOG performance status, and IL-6 levels, are detailed in Table 3.

### 3.4. Progression-Free Survival (PFS) and Overall Survival (OS)

**Figure 1:**
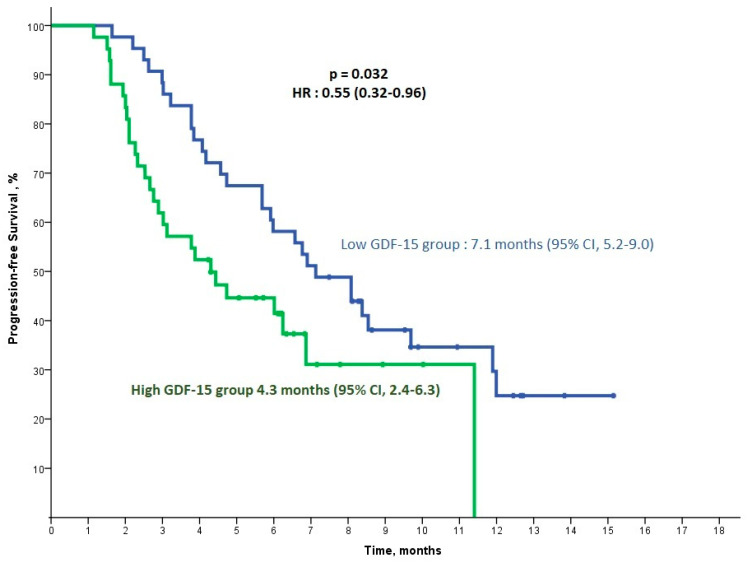
Kaplan–Meier progression-free survival analysis for the low/high-GDF-15 group. This analysis includes the final cohort of 85 patients after excluding 12 patients for irregular follow-up visits.

**Figure 2:**
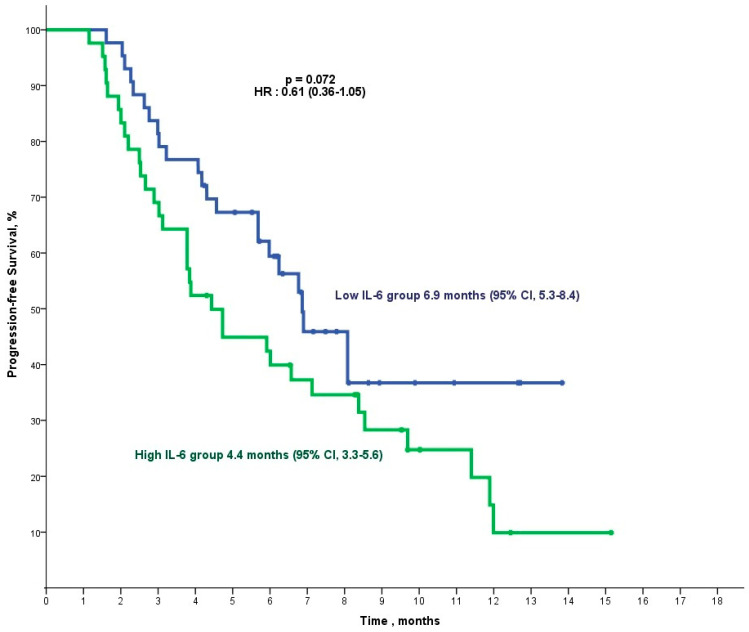
Kaplan–Meier progression-free survival analysis for the low/high-IL-6 group. This analysis includes the final cohort of 85 patients after excluding 12 patients for irregular follow-up visits.

The median follow-up duration in our study was 13.4 months. Patients in the low-GDF-15 group demonstrated a significantly longer median progression-free survival (mPFS) of 7.1 months (95% CI, 5.2–9.0) compared with 4.3 months (95% CI, 2.4–6.3) in the high-GDF-15 group (*p* = 0.032, HR: 0.55 (0.32–0.96)) (Figure 1). For IL-6, patients in the low-IL-6 group had an mPFS of 6.9 months (95% CI, 5.3–8.4), while those in the high-IL-6 group exhibited a shorter mPFS of 4.4 months (95% CI, 3.3–5.6), though this difference was not statistically significant (*p* = 0.072, HR: 0.61 (0.36–1.05)) (Figure 2).

**Figure 3:**
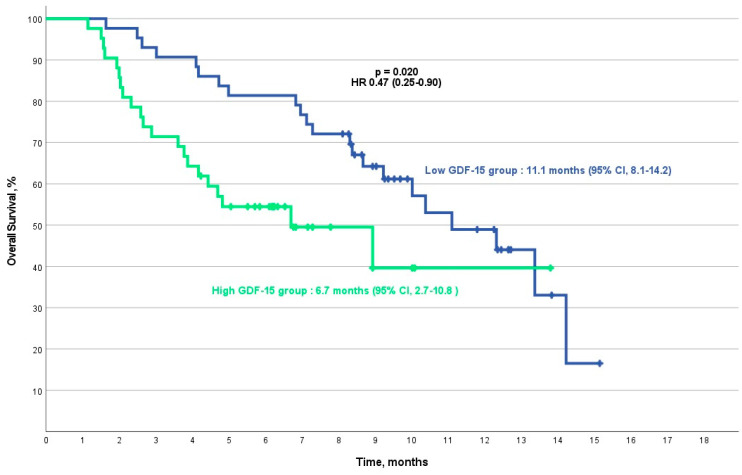
Kaplan–Meier overall survival analysis for the low/high-GDF-15 group. This analysis includes the final cohort of 85 patients after excluding 12 patients for irregular follow-up visits.

The median overall survival (mOS) for the entire cohort was 10.0 months (95% CI, 7.9–12.1). Patients in the low-GDF-15 group had an mOS of 11.1 months (95% CI, 8.1–14.2), compared with a significantly shorter mOS of 6.7 months (95% CI, 2.7–10.8) in the high-GDF-15 group. This difference in overall survival was statistically significant (*p* = 0.020), with a hazard ratio of 0.47 (95% CI, 0.25–0.90) (Figure 3).

**Figure 4:**
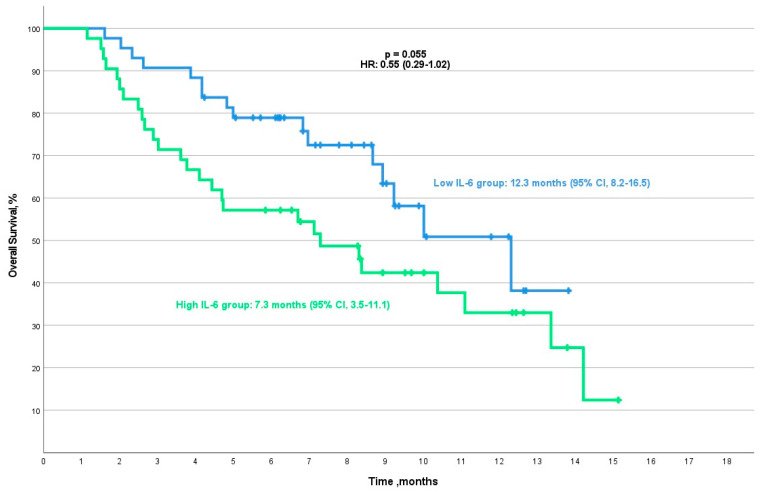
Kaplan–Meier overall survival analysis for the low/high-IL-6 group. This analysis includes the final cohort of 85 patients after excluding 12 patients for irregular follow-up visits.

Patients in the low-IL-6 group had an mOS of 12.3 months (95% CI, 8.2–16.5), while those in the high-IL-6 group exhibited an mOS of 7.3 months (95% CI, 3.5–11.1). Although a trend toward longer survival was observed in the low-IL-6 group, the difference between the two groups did not reach statistical significance (*p* = 0.055, HR: 0.55 (95% CI, 0.29–1.02)) (Figure 4).

### 3.5. NSCLC Subgroup Progression-Free Survival Analysis Based on GDF-15 Levels

**Figure 5:**
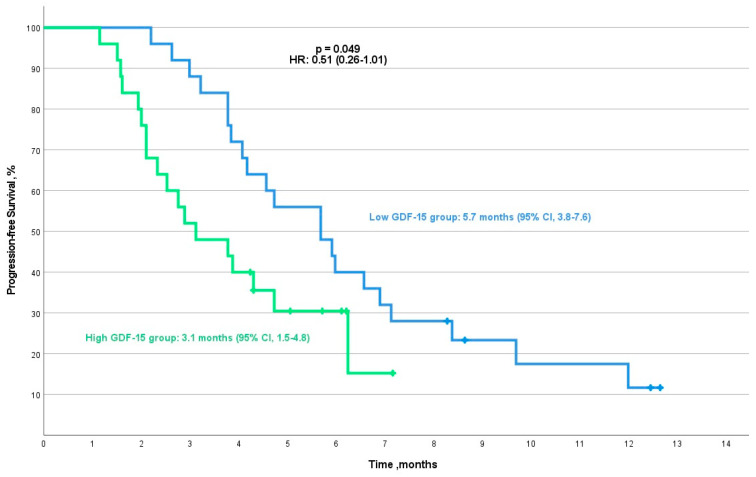
Kaplan–Meier curve for progression-free survival (PFS) in NSCLC patients by low/high-GDF-15 group.

**Figure 6:**
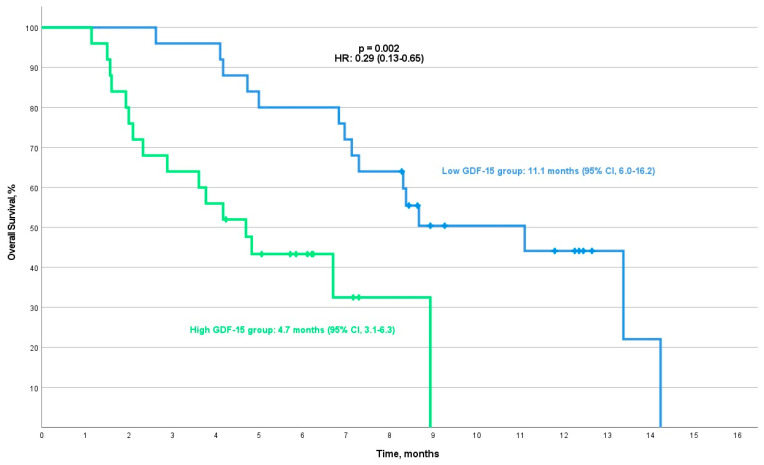
Kaplan–Meier curve for progression-free survival (PFS) in the low/high-GDF-15 group of NSCLC patients.

In NSCLC, the median progression-free survival (mPFS) for patients in the low-GDF-15 group was 5.7 months (95% CI, 3.8–7.6), compared with 3.1 months (95% CI, 1.5–4.8) for those in the high-GDF-15 group. This difference in mPFS approached statistical significance (*p* = 0.049, HR: 0.51 (95% CI, 0.26–1.01)) (Figure 5). The median overall survival was 11.1 months (95% CI, 6.0–16.2) for the low-GDF-15 group, compared with 4.7 months (95% CI, 3.1–6.3) for the high-GDF-15 group (*p* = 0.002, HR: 0.29 (95% CI, 0.13–0.65)) (Figure 6).

### 3.6. Univariate and Multivariate Cox Regression Analyses of Progression-Free Survival (PFS)

The univariate Cox regression analysis revealed that the low-GDF-15 group was significantly associated with improved progression-free survival (PFS) compared with the high-GDF-15 group (HR 0.553, 95% CI 0.319–0.959, *p* = 0.032).

In the multivariate analysis, low GDF-15 levels remained an independent predictor of longer PFS (HR 0.362, 95% CI 0.196–0.669, *p* = 0.001). The absence of cachexia (HR 0.447, 95% CI 0.233–0.857, *p* = 0.015) and the cancer type (NSCLC: HR 2.941, 95% CI 1.620–5.340, *p* < 0.001) were also significant predictors. Other variables, such as age and IL-6 levels, did not retain statistical significance in the multivariate model. These findings are summarized in Table 4.

### 3.7. Univariate and Multivariate Cox Regression Analyses of Overall Survival (OS)

The univariate Cox regression analysis showed that patients in the low-GDF-15 group had significantly longer OS compared with those in the high-GDF-15 group (HR 0.473, 95% CI 0.249–0.901, *p* = 0.020).

In the multivariate analysis, low GDF-15 levels remained an independent predictor of improved OS (HR 0.296, 95% CI 0.145–0.602, *p* = 0.001). The cancer type (NSCLC: HR 2.461, 95% CI 1.235–4.904, *p* = 0.010) was also a significant predictor. Other variables, such as cachexia, age, and IL-6 levels, did not retain statistical significance in the multivariate model. These findings are summarized in Table 5.

### 3.8. Cachexia Status and Serum GDF-15 Levels

Cachexia was observed in 43% (*n* = 18) of patients with high GDF-15 levels, compared with only 21% (*n* = 9) in those with low GDF-15 levels (*p* = 0.037). This suggests a statistically significant association between elevated GDF-15 levels and the development of cancer-related cachexia. No significant associations were found between IL-6 levels and cachexia development (*p* = 0.130).

## 4. Discussion

The present study evaluated the prognostic significance of serum GDF-15 and IL-6 levels in patients undergoing immunotherapy for advanced malignancies, including NSCLC, RCC, and malignant melanoma. Our findings reveal that elevated serum GDF-15 levels are significantly associated with shorter mPFS and mOS, as well as an increased risk of developing cancer-associated cachexia. Although IL-6 was also correlated with PFS, its predictive capacity appeared less robust in comparison to GDF-15.

The association between elevated GDF-15 levels and adverse clinical outcomes aligns with prior studies identifying GDF-15 as a key mediator of immune evasion and tumor progression across various cancers. GDF-15 contributes to an immunosuppressive tumor microenvironment by inhibiting T-cell activation, which may reduce the effectiveness of immune checkpoint inhibitors [17,18]. This mechanism likely explains the negative impact of high GDF-15 levels on both PFS and OS in our cohort. Previous studies in metastatic melanoma patients treated with ipilimumab have shown that elevated serum GDF-15 levels adversely affect overall survival, suggesting that this association may reflect disease characteristics rather than a direct impact on immunotherapy response [19]. Our study is the first to specifically assess the relationship between serum GDF-15 levels and outcomes in patients receiving anti-PD-1 therapy. Additionally, while our past research has linked tumor GDF-15 expression to nivolumab response in NSCLC [20], the findings of the current trial emphasize the clinical importance of serum GDF-15 as a dynamic biomarker reflecting individual patient characteristics. Our study also supports previous findings connecting elevated GDF-15 levels with cancer cachexia, a condition that severely diminishes patients’ functional status and quality of life [18,21]. GDF-15 has been identified as a major factor in cancer cachexia, promoting muscle wasting and anorexia through the MIC-1/GDF-15-GFRAL signaling pathway [17].

The role of IL-6 in cancer immunotherapy remains a topic of ongoing debate. While our results demonstrate a negative correlation between IL-6 levels and PFS, this association was less pronounced than that observed for GDF-15. The pro-inflammatory nature of IL-6, which has been implicated in various tumor-promoting processes, suggests that its elevated levels could contribute to systemic inflammation and, in turn, reduced efficacy of immunotherapeutic interventions [9]. However, the conflicting findings in the literature regarding IL-6′s role in immunotherapy response suggest that further research is necessary to clarify its prognostic utility [10,22].

The clinical implications of these findings are noteworthy. Given the increasing use of immunotherapy in diverse oncologic settings, particularly in resource-constrained environments, the identification of reliable biomarkers such as GDF-15 could guide clinical decision making. By stratifying patients based on biomarker levels, it may be possible to prioritize those who are most likely to benefit from immunotherapy, thereby optimizing treatment outcomes and reducing healthcare costs. Moreover, GDF-15′s association with cancer cachexia suggests that it may serve as a potential therapeutic target. Emerging evidence, including recent trials investigating GDF-15 inhibitors such as ponsegromab, indicates that targeting GDF-15 could alleviate cachexia symptoms and improve patient quality of life [21]. These therapeutic strategies warrant further investigation, particularly in combination with immunotherapy.

Despite the strengths of this study, several limitations should be noted. While the sample size was sufficient for exploratory analyses, it may affect the generalizability of the findings. Additionally, nivolumab was administered as second-line monotherapy due to healthcare policies in our country, which could have influenced patient outcomes. Biomarker levels were only assessed at baseline, limiting the ability to evaluate dynamic changes in GDF-15 and IL-6 during treatment. Future studies should include serial measurements of these biomarkers to better understand their role in monitoring treatment response.

## 5. Conclusions

In conclusion, elevated serum GDF-15 levels were identified as a significant predictor of shorter PFS and OS and an increased risk of cachexia in patients receiving immunotherapy for advanced cancers. While IL-6 showed some prognostic value, GDF-15 emerged as a more robust biomarker. These findings suggest the potential of GDF-15 as a prognostic biomarker for immunotherapy response and a possible therapeutic target, particularly in patients at risk of developing cachexia. Further research is needed to confirm these results in larger, multicenter cohorts and to better understand the role of GDF-15 in the context of immunotherapy.

## Figures and Tables

**Table 1:** Patient characteristics and biomarker levels.

Characteristic	*n* = 85 (%)
Sex	
- Male	58 (68%)
- Female	27 (32%)
Median age (years)	64.5 (34–80)
Cancer type	
- NSCLC	50 (59%)
- RCC	23 (27%)
- Malignant melanoma	12 (14%)
ECOG performance status	
- ECOG 0	31 (36%)
- ECOG 1	54 (64%)
Biomarker levels, median (range)	
- GDF-15 (ng/mL)	26.4 (14.7–798.9)
- IL-6 (pg/mL)	20.0 (1.0–183.3)

**Table 2:** Spearman correlation coefficients (ρ) for biomarkers and PFS.

Variable	GDF-15 (ρ, *p*-Value)	IL-6 (ρ, *p*-Value)	mPFS (ρ, *p*-Value)
GDF-15	1.000	−0.063, *p*: 0.569	−0.416, *p* < 0.001
IL-6	−0.063, *p* = 0.569	1.000	−0.257, *p* = 0.018
mPFS	−0.416, *p* < 0.001	−0.257, *p* = 0.018	1.000

**Table 3:** Baseline characteristics of patients by GDF-15 level.

Characteristic	Low GDF-15 (<26.4 ng/mL)	High GDF-15 (≥26.4 ng/mL)
Number of patients	*n* = 43	*n* = 42
Cancer type		
- NSCLC (%)	25 (58%)	25 (59%)
- RCC (%)	10 (23%)	13 (31%)
- Malignant melanoma (%)	8 (19%)	4 (10%)
Sex		
- Male (%)	29 (67%)	29 (69%)
- Female (%)	14 (33%)	13 (31%)
ECOG performance status		
- ECOG 0 (%)	15 (35%)	16 (38%)
- ECOG 1 (%)	28 (65%)	26 (62%)
Median IL-6 level (pg/mL)	20.1 (1.8–183.3)	19.6 (1.0–116.3)

**Table 4:** Univariate and multivariate analyses for progression-free survival (PFS).

Variable	Univariate Analysis (HR, 95% CI, *p*-Value)	Multivariate Analysis (HR, 95% CI, *p*-Value)
GDF-15 group		
- Low group	**0.553 (0.319–0.959),** ***p* = 0.032**	**0.362 (0.196–0.669),** ***p* = 0.001**
- High group (reference)	1.00	1.00
Cachexia		
- No	0.636 (0.309–1.166), *p* = 0.054	**0.447 (0.233–0.857),** ***p* = 0.015**
- Yes (reference)	1.00	1.00
Age		
- <65	0.614 (0.360–1.047), *p* = 0.070	0.653 (0.379–1.126), *p* = 0.126
- ≥65 (reference)	1.00	1.00
IL-6 group		
- Low group	HR: 0.612 (0.358–1.045) *p* = 0.072	0.677 (0.388–1.181), *p* = 0.169
- High group (reference)	1.00	1.00
Cancer type		
- NSCLC	**2.531 (1.417–4.523),** ***p* = 0.002**	**2.941 (1.620–5.340)** ***p* < 0.001**
- Other (reference)	1.00	1.00

**Table 5:** Univariate and multivariate analyses for overall survival (OS).

Variable	Univariate Analysis (HR, 95% CI, *p*-Value)	Multivariate Analysis (HR, 95% CI, *p*-Value)
GDF-15 group		
- Low group	**0.473 (0.249–0.901),** ***p* = 0.020**	**0.296 (0.145–0.602),** ***p* = 0.001**
- High group (reference)	1.00	1.00
Cachexia		
- No	0.544 (0.260–1.140), *p* = 0.089	**0.445 (0.204–0.968),** ***p* = 0.041**
- Yes (reference)	1.00	1.00
Age		
- <65	0.700 (0.379–1.292), *p* = 0.251	
- ≥65 (reference)	1.00	
IL-6 group		
- Low group	HR: 0.547 (0.292–1.024) *p* = 0.055	0.569 (0.298–1.085), *p* = 0.087
- High group (reference)	1.00	1.00
Cancer type		
- NSCLC	**2.288 (1.170–4.473),** ***p* = 0.011**	**2.461 (1.235–4.904)** ***p* = 0.010**
- Other (reference)	1.00	1.00

## Data Availability

The data presented in this study are available upon request from the corresponding author. The data are not publicly available due to privacy concerns and institutional regulations governing patient confidentiality.
